# Adverse Health Effects and Mercury Exposure in a Colombian Artisanal and Small-Scale Gold Mining Community

**DOI:** 10.3390/toxics10120723

**Published:** 2022-11-25

**Authors:** Fredy Vergara-Murillo, Shirley González-Ospino, Nazly Cepeda-Ortega, Fredy Pomares-Herrera, Boris Johnson-Restrepo

**Affiliations:** 1 Environmental Chemistry Research Group, School of Exact and Natural Sciences, University Campus of San Pablo, University of Cartagena, Zaragocilla, Carrera 50 No. 24-99, Cartagena 130015, Colombia; 2 School of Medicine, University of Cartagena, Zaragocilla, Carrera 50 No. 24-99, Cartagena 130015, Colombia; 3 Synergia, Health Provider Institution, Cartagena 130005, Colombia

**Keywords:** mercury, artisanal small-scale gold mining, miners, lung function impairment, neurotoxicity effects, occupational exposure

## Abstract

The aim of this study was, first of all, to associate the mercury (Hg) concentrations and respiratory functions of the gold miners in the artisanal small-scale gold mining (ASGM) environment in San Martín de Loba, Colombia. We carried out a cross-sectional study using a survey whereby we collected basic demographic information, occupational medical history, and applied two validated questionnaires (Q16 and SF36). We measured Hg levels in all volunteers using direct thermal decomposition-atomic absorption spectrometry. Univariate and bivariate statistical analyses were carried out for all variables, performing logistic regression to assess the effect of ASGM on health outcomes. Volunteers enrolled (n = 124) were between the ages of 20 and 84 years (84% miners and 79% males). No changes were found in the systolic blood pressure, diastolic blood pressure, and heart rate from the ASGM miners, in crude and adjusted statistical analyses. ASGM miners increased 8.91 (95% confidence interval, 1.55–95.70) times the risk of having these than of having neurotoxic effects. Concentrations of total whole blood mercury (T-Hg) in all participants ranged from 0.6 to 82.5 with a median of 6.0 μg/L. Miners had higher T-Hg concentrations than non-miners (*p*-value = 0.011). Normal and abnormal respiratory spirometry patterns showed significant differences with the physical role and physical function of quality-of-life scales (the (*p*-value was 0.012 and 0.004, respectively). The spirometry test was carried out in 87 male miners, with 25% of these miners reporting abnormalities. Out of these, 73% presented a restrictive spirometry pattern, and 27%, an obstructive spirometry pattern. The ASGM population had higher Hg concentrations and worse neurotoxic symptomatology than non-miners of the same community.

## 1. Introduction

Artisanal and small-scale gold mining (ASGM) directly and indirectly employs more than 100 million people in over 70 countries around the world [1,2]. An estimated 10–15 million people work directly on ASGM activities worldwide, out of which 4–5 million are women and children [3,4,5] and this is increasing in Latin America every year. ASGM produces 12 to 15% of the gold in the world,, yet it uses more than 1400 tons/year of elemental mercury (Hg) which is released in the air, water, sediments, land and food [3]. Elemental mercury released into bodies of water after ASGM activities can be oxidized into soluble species (Hg^2+^), which can then be deposited in the sediments and converted by natural process into methylmercury (MeHg), the most toxic of the Hg species. The MeHg can be bioaccumulated and biomagnified in fish via the food web, and then eaten by the human population [4,5,6].

ASGM is characterized as an informal job that requires limited technical skills and few resources, but requires the use of hazardous substances, such as elemental Hg for gold amalgamation. Mercury contamination has frequently been associated to a set of adverse health effects and environment impacts with social consequences [7,8]. These ASGM miners do not normally use any required personal protective equipment (including masks, gloves, helmets or boots). People in the areas near mining sites, including children, pregnant women and the elderly, are also exposed. Pathways to mercury exposure for ASGM miners include dermal contact and the inhalation of vapor during the amalgamation, inhalation after amalgam smelting to isolate the gold, as well as eating contaminated fish [9,10,11].

Recent studies have show that mercury is a powerful neurotoxic and induces serious health effects, such as irreversible neurological disorders, damage to the bone system, teratogenicity, oxidative stress, hormonal alterations, kidney injury, hearing loss, endocrine effects, infertility, menstrual alterations, spontaneous abortions, and other adverse health conditions [9,11,12,13,14,15,16,17]. Additionally, elemental Hg and its inorganic salts are corrosive to the skin, eyes, and gastrointestinal tract by ingestion [18]. Over the last decade, we have increased our understanding of the critical processes involved in the global cycle of Hg and how this element moves in the environment. ASGM is one of the largest emitters of Hg in freshwater globally, releasing approximately 880 tons every year [19]. In many places, the use of mercury may be restricted and its use in ASGM is illegal. Releasing Mercury from an ASGM is considered a risk that affects the health of miners, their communities, and the environment. Therefore, mercury pollution is expected to be reduced in the future in anthropogenic activities responsible for global emissions with the implementation of the international agreement of the Minamata Convention on Mercury, adopted by 140 countries [20].

In some countries, the precious metal in ASGM is extracted from ores containing gold, in which rocks are finely pulverized (i.e., sinkholes) by hand with hammers and later using ball mills. The material reduced to powder is mixed with elemental Hg forming an amalgam (a gold–Hg alloy) [19]. During the rock pulverization process, dust emissions producing particulate matter (PM) can be dispersed in the air. Exposure to PM is associated with lung function impairment, depending on PM size and its composition. The vast majority of PM from mining activities are formed from particles greater than PM 10, generated from mechanical activities on the rock and dispersed by the wind and suspended in the air. Exposure to airborne aerosols as small (PM2.5 and PM10) or larger particles (more than PM10) can lead over time to be absorbed through the lungs and associated with respiratory diseases that can affect the health of workers, including symptoms such as coughing, increasing breathlessness, and respiratory pathologies such as asbestosis, mesotheliomas, pneumoconiosis, tuberculosis, or lung cancer [21]. 

Colombia reported between 268 and 418 thousand people working as ASGM miners, who lost a combined total of 23–52 thousand years of life [22]. In San Martin de Loba (Bolivar), a small municipality in Colombia, sinkhole ASGM has been carried out since the pre-Hispanic era [23]. Approximately 70% of the 17,295 inhabitants of San Martin de Loba depend on ASGM activities for income [8,24]. Several studies on ASGM in Latin America have shown Hg levels in air, food, and biological samples that exceed the critical threshold of the Agency for the Registration of Toxic Substances and Diseases of the Centers for Disease Control and Prevention of the United States [24,25] but few studies have focused on lung function impairments associated with gold mining [26]. The main routes of mercury poisoning are metal vapors, yet the exposure by inhalation of particles containing species of this element is very low. The threshold limit for T-Hg in whole blood according to the World Health Organization is 10 μg/L, whereas it is 5 μg/L for the Environmental Protection Agency [27,28,29].

Miners working in ASGM in San Martin de Loba (Bolivar, Colombia) are exposed to mercury through the extraction of gold from excavations of sinkhole mines which are linked to health hazards related to lung function impairment, chronic lung disease, and cognitive dysfunction, in terms of neurological effects. Therefore, the aim of this study was to determine lung function, total Hg levels in whole blood, neurotoxicity (Q16) and quality of life (SF-36) in ASGM miners and non-miners from this municipality [30,31].

## 2. Materials and Methods

### 2.1. Design and Study Site

**Figure 1:**
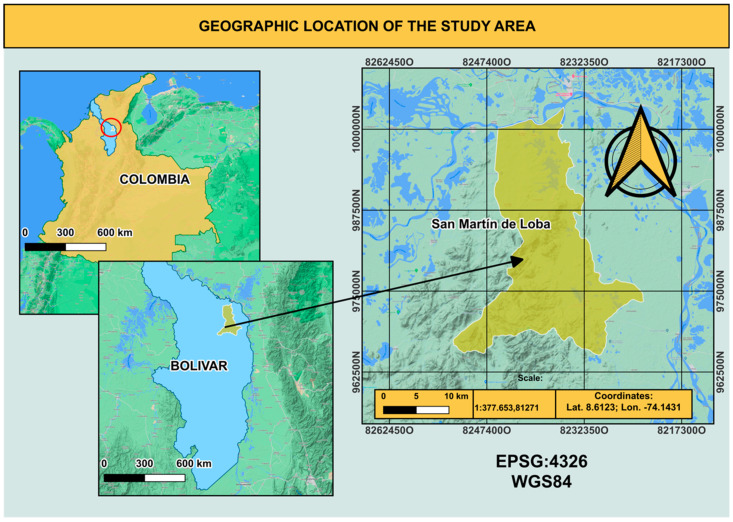
Location of San Martin de Loba (Bolívar, Colombia).

A cross-sectional study was designed and 124 volunteers of both sexes were enrolled in November 2018, including 98 men and 26 women, aged 20–84 and 25–71, respectively. With the approval of the authorities and miners’ associations of the municipality of San Martin de Loba, volunteers who met the study’s selection criteria were invited to participate. People were eligible if they had been living for at least two years in San Martin de Loba, Colombia. This municipality is located in northern Colombia (8°56′12″ N, 74°2′20″ W) at an altitude of 30 m above sea level with an average temperature of 27 °C and an extension of 414 km^2^ (Figure 1). The selection criteria for the analysis included miners and non-miners (housekeepers, cooks, miner assistants, and additional participants involved in other activities). After reading the entire written informed consent to explain the purpose of the study, all participants were free to ask questions then they signed the consent form. Before sampling, the institutional ethical review board of the University of Cartagena approved this study by minute No. 75, 2014.

### 2.2. Demography and Health Surveys

Participants completed a brief written survey so we could collect basic demographic information. Occupational medical history and two validated questionnaires (Q16 and SF36) were applied to evaluate the quality of life and the possible neurological impacts on the participants. Health-related quality of life (SF-36) is a standardized questionnaire of 36 items grouped into 8 measurable scales: physical functioning (PF), physical role (PR), bodily pain (BP), general health (GH), vitality (VT), social functioning (SF), emotional role (RE) and mental health (MH) [30]. Each dimension is categorized from 0 to 100%, from worst to best quality of life. A standardized questionnaire (Q16) was performed to screen patients with possible neurotoxic prevalence symptoms among the exposed population to hazardous substances such as Hg [28]. If the participant reported more than six negative symptoms in the Q16, the patient was considered as requiring evaluation by a health professional for possible neurotoxic effects [31,32].

### 2.3. Spirometry as a Performed Pulmonary Function Test (PFT)

The spirometry test was applied to the miners involved in this study. Miners working in sinkholes are exposed to PM and may display respiratory conditions. Volunteers who completed the medical history and health surveys underwent a spirometry test using the SpiroBank II (MIR, Roma-Italy) [21]. Spirometry is a breathing test to measure the amount of air volume (ml) that individuals can blow out of their lungs. It is considered a performed pulmonary function test (PFT) and is used widely to be simple and reproducible. A spirometry test is useful to diagnose different types of pulmonary disorders. The PFT took approximately 15 min. The subjects performed three acceptable maneuvers in which the two best measurements of forced vital capacity (FVC) and forced expiratory volume in one second (FEV1) differ by less than 150 mL between them. The result was chosen from the curve in which the sum of the FVC and FEV1 is higher. An obstructive pattern defect on spirometry was indicated if an FEV1/FVC ratio was less than 70%, while a restrictive pattern defect was present at low FVC in the presence of a normal FEV1/FVC ratio.

### 2.4. Blood Sample Collection

A blood sample of 4 mL was extracted by venipuncture and collected in vacutainer^®^ tubes with EDTA as an anticoagulant (Becton Dickinson, UK, Oxford), which were labeled with the information of the subjects. Tubes with whole blood samples were transported to the laboratory in a refrigerated container at 4 °C and stored at −20 °C until analysis. We organized the blood samples with a consecutive code for each volunteer, avoiding the possibility that any researcher could identify the results of any person in the population. Only medical personnel interviewing patients knew the identity of each subject.

### 2.5. Instrumental Analysis

The determination of T-Hg concentrations in blood samples was tested in whole blood samples using a thermal decomposition atomic absorption spectrometer (TD-AAS) with Zeeman-effect background correction technique, RA-915M equipped with pyrolysis accessory, PYRO 915+ for solid sample analysis (Lumex Instruments, St. Petersburg, Russia). T-Hg concentrations were measured directly in the whole blood samples without any digestion or sample preparation before analysis. Blood samples (~100 mg) were weighed into a quartz boat and heated at 800 °C to complete combustion in order to release the Hg gas to be measured based on the absorbance at a wavelength of 253.7 nm in the enclosed system. After thermal release, the quantitative signal for Hg is shown as the total area under the peak. Before every measurement, the quartz boat was cleaned and heated once again to obtain the instrument baseline.

### 2.6. Quality Assurance and Quality Control

A series of analytical quality assurance and quality control procedures were carried out in order to maintain the traceability of the results of mercury concentrations. Each sample was analyzed in duplicate; if the coefficient of variation (CV) was above 20%, a confirmation reading was carried out. The limit of detection (LOD) and limit of quantification (LOQ) were 1.4 and 4.36 µg/L, respectively. calibration curves were created directly using whole blood Standard Reference Materials (SRM) with known mercury concentrations provided by the Wadsworth Center, New York State Department of Health [33]. For this study, the instrument was calibrated using the SRMs BE12-12, BE12-14, and BE 12-15, corresponding to three different concentrations of 2.39 µg/L, 3.21 µg/L and 11.35 µg/L, respectively. Different masses of the SRMs were weighted in a quartz boat to create a five-point calibration curve (3, 7, 9, 34 and 42 µg/L). The accuracy and precision of the method were obtained by measuring five times the BE12-12, BE12-14, and BE12-15 CRMs with accuracies of 97.1% (2.32 ± 0.22 µg/L), 87.2% (2.8 ± 0.29 µg/L) and 109% (12.37 ± 0.64 µg /L), respectively. For all cases, the precisions of SRMs as %CV were < 5%. Calibration curves were obtained with a regression coefficient ≥ 0.995. The calibration curves were created on a daily basis with the SRMs, verifying measurement with an SRM every ten times as a control during the analysis.

### 2.7. Statistical Analysis

Univariate and bivariate statistical analyses were carried out for all variables. The normal distribution of data was assessed using the Kolmogorov–Smirnov one sample test. Due to the lack of normality of some continuous variables as T-Hg concentrations, the Wilcoxon-Mann-Whitney test (WMW) was used to compare only two groups, whereas the Kruskal–Wallis test was used to find the difference between three or more independent groups. Possible associations between T-Hg concentrations and the independent variables were separately explored using the Spearman correlation analysis. Due to the uncertainties in exposure measures and the response to individual variability, the precise minimum concentration of mercury in the blood that may cause toxic effects in humans could not be determined. However, it can be used as a cut-off concentration in the whole blood of 5 μg/L based on the US-EPA reference dose (RfD) [27]. The population of miners was divided into two subgroups as dichotomous outcomes in descriptive analyses: participants with a low exposure (≤5 μg/L of Hg in the blood) made up the first group and those with high-exposure doses (>5 μg/L of Hg in the blood) formed the second group. 

A logistic regression analysis was used to assess the effect of mining exposure on the items of the Q16 questions. In the multivariable model the confounder variables included were age, sex, systolic blood pressure, diastolic blood pressure, heart rate, and body-mass index. Non-significant variables in crude analyses were deemed non-confounders and were not included in the final model. The body-mass index entered the model as a second-degree polynomial to account for non-linearity. We report adjusted odds ratios (OR) with 95% confidence intervals (CI). All comparisons among groups or correlations were presented a level of significance of *p* ≤ 0.05. All statistical analyses were calculated with R, version 4.2.1 (R Core Team, 2022).

## 3. Results

### 3.1. Population Characteristics

**Figure 2:**
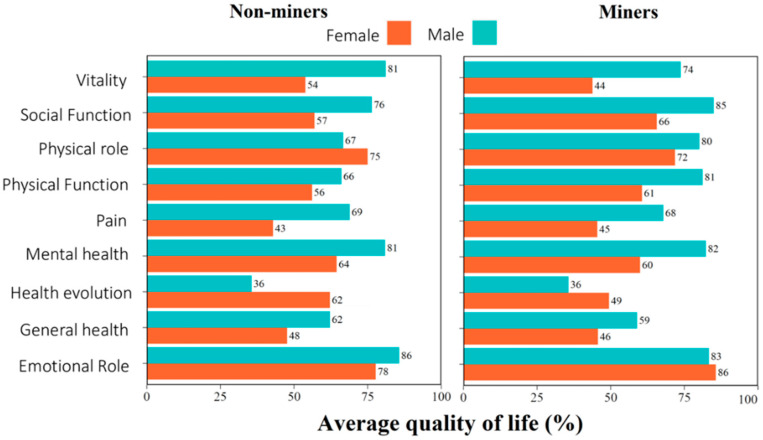
Average quality of life in miners and non-miners, stratified by sex.

The study was conducted among 124 participants who signed the written informed consent and completed the full questionnaires. The participants’ sociodemographic characteristics are summarized in Table 1. The participants were 84% miners and 79% males (Table 1). The average of participants was 46.1 ± 13.0 (mean ± SD), ranging from 20 to 84 years, where miners had a mean age of 45.5 ± 12.4, and non-miners, of 49.3 ± 16.2 years. There were no statistical differences between the ages of the participants of both groups (*t*-test, *p* = 0.26). A total of 46% of the participants had attended elementary school; and 38%, middle/high school; and 5% had obtained an undergraduate degree. On average, participants had a body-mass index (BMI) of 24.3 ± 3.9 and ranged from 16.9 to 32.3 kg/m^2^. Ten percent of participants surveyed were overweight (BMI ≥ 30). No ASGM miners were found to have changes in systolic blood pressure, diastolic blood pressure, and heart rate with crude and adjusted analyses (Table 2). A total of 80% of the entire female population (miners and non-miners) reported joint pain; 43%, fatigue; 53% had undergone one or more abortions; and 26% reported irregular menstrual bleeding. The quality of life in male and female miners is shown in Figure 2.

### 3.2. Concentrations of T-Hg

**Figure 3:**
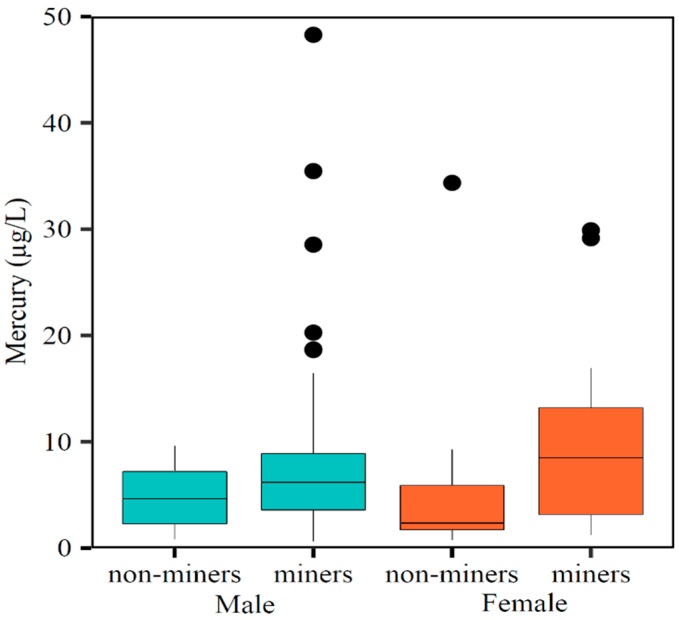
Box-plot of whole blood T-Hg concentrations in miners and non-miners, stratified by sex, indicating variability outside the upper and lower quartiles. Note: One outlier with a whole blood T-Hg level value >50 µg/L was excluded from the box plot (a male exposed to mining).

Concentrations of T-Hg in all participants presented a median of 6.0 μg/L and ranged from 0.6 to 82.5 μg/L. Miners had higher T-Hg concentrations than non-miners, with a statistical difference between the two groups in which the median of T-Hg concentrations for the miners was twice as high as those for the non-miners (median: 6.2 versus 3.1 μg/L, *p*-value = 0.011) (Table 1 and Figure 3). Concentrations of T-Hg in women miners were not statistically different (*p*-value of 0.06). A total of 58.9% of all the participants had higher concentrations of T-Hg than the environmental exposure (>5 μg/L) and 10.4% for higher occupational exposure (≥15 μg/L).

### 3.3. Neurotoxicity

Neurotoxicity factors obtained from the miners were associated to Hg exposure (low and high) (Table 2). ASGM activities are related to increased neurotoxicity, including loss of understanding, problems with usual activities, fatigue, oppression in chest, feeling of falling down when standing, painful tingling, loss of strength in extremities and loss of sensitivity in arms/legs (Table 3). In addition, miners in ASGM increased the risk of having more than 6 neurotoxic abnormalities by 8.9 times. The logistical regression analysis was adjusted to demographic factors (age and sex), Hg exposure, BMI, and occupation (Table 3) [34].

### 3.4. Quality of life (Sf-36)

For participants, the lowest quality of life was noted in the physical role (50%) and general health (57%), although the highest quality of life was social function (87.5%) and emotional role (86%). Both mining and non-mining women had a lower quality of life than men (Figure 2). The physical role and physical function of the quality of life scales were different between normal and abnormal respiratory spirometry patterns (*p* < 0.05). The physical function, pain, general health, vitality, social function, emotional role and mental health were different between Low and High mercury concentrations in the male and female populations (*p* < 0.05) (Table 4). 

### 3.5. Spirometry Test and Lung Function in Miners

The results of the spirometry test were classified in different patterns according to lung function such as normal, abnormal, obstructive, and restrictive spirometry. The patterns of the spirometric test were associated with the results of quality of life (SF-36) for a better intepretation (Table 5). The 87 male miners evaluated had the following lung functions: 25% abnormality, 73% restrictive, and 27% obstructive spirometry patterns (Table 5). 

### 3.6. Neurotoxic Symptoms (Q16)

Neurotoxic symptoms were measured in 124 participants, out of which 58.2% reported at least 6 negative responses. Table 3 shows that 57% of the miners had high levels of mercury in their blood (Hg ≥ 5 μg/L) against 45.5% of the miners with low levels of mercury in their blood (Hg < 5 μg/L). In women, 84.6% showed 6 more neurotoxic symptoms compared to 51.5% of men (Table 3).

## 4. Discussion

Recent studies in Latin America on ASGM workplaces have shown high mercury concentrations in biological samples (hair, blood, and urine) taken from miners, due to the use of mercury [26,34,35,36,37,38,39,40,41,42] (Table 6). Several studies have shown a significant impact of the use of Hg on the environment and the health of miners and people living in the vicinity of ASM miners [8,25,36]. Reported concentrations of Hg in the environment increase in ASGM sites. Most of these studies were published in Colombia, Peru, Ecuador and Mexico, displaying the rise of ASGM in Latin America and the lack of knowledge regarding Hg management in extractive processes. Although women are part of this practice, the number of women surveyed is generally low in these recent studies (Table 6). 

Our results concurred with the findings of previous studies. Mean concentrations of T-Hg in blood samples of the miners from San Martin de Loba were similar or greater to values from miners or communities near ASGM in other studies in Colombia [43,44,45,46], yet lower than those reported by Cruz-Esquivel et al., 2019 [26] and Calao-Ramos et al., 2021 [45] (Table 6). In general, mean concentrations of T-Hg in blood samples taken from female miners in this study were greater than those from male miners, although there were no significant statistical differences between them. The lack of statistical difference between T-Hg concentrations of males and females may be due to the small sample size in this study. This result was similar to other studies carried out in Colombia [26,43,45] (Table 6). The results for quality of life (SF-36) were adjusted at low and high T-Hg concentrations; therefore, statistical differences were found between females and males in 7 of the 8 evaluated parameters (Table 4). 

The largest concentration of Hg in whole blood was found in ASGM in departments such as Choco [41], a poor region in this country, similar to San Martin de Loba (the present study). These socioeconomic inequalities are correlated with ASGM [4,39] and have the potential to interact with Hg by reducing the opportunity for health care for the consequences of toxicity, with lower education levels. In addition, some of these settings are more susceptible to illegal mercury trade, and violence is used to control the territory. These three factors—Hg toxicity, poverty, and violence—are synergically interacting with each other and increasing the adverse effects of Hg on these populations and the environment [47,48]. 

The ASGMs have been considered hazardous working places for miners because they involve rudimentary and semi-automatic tools, and miners are usually associated with poor health without following the safety standards such as wearing personal protection elements (PPE) that could include gloves, safety glasses, shoes, earplugs, hats, respirators, or body suits, etc. Miners from ASGMs in San Martin de Loba do not wear PPE or they have poor PPE compliance, greatly increasing the risk of mercury exposure. Therefore, these miners had high mercury concentrations in blood samples because they have been directly exposed to mercury vapor produced during the amalgamation and the burning processes, as well as eating fish that has been contaminated with methylmercury. Previous studies report that the exposure of miners to mercury in order to extract gold can cause immune, sensory, neurological, motor, and behavioral dysfunctions similar to neuronal diseases [32,33]. In a systematic review of recent studies reporting Hg concentrations in male and female miners from ASGMs in Latin America, Colombia is the country with the most publications, followed by Mexico, Ecuador, and Peru (Table 5).

**Figure 4:**
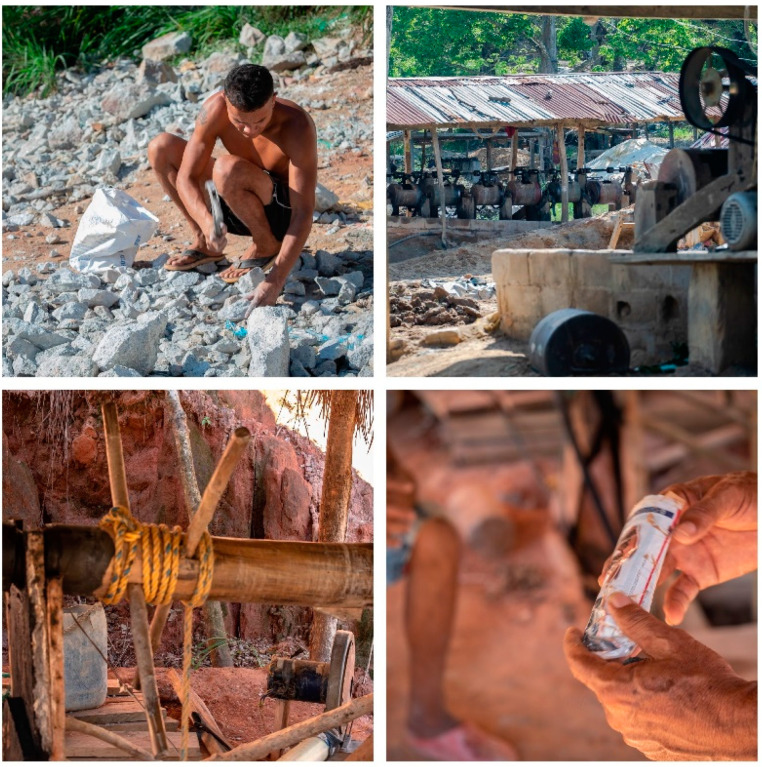
**Top left**, a miner reduces the particle size of the raw minerals extracted from the mine; **top right**, the mills; **bottom left**, the rudimentary system with which it settles up to 40 m, turning the crank manually; in the same quadrant we see an impromptu ventilation system used to exchange air in the tunnels; **bottom right**, a paper cartridge used to build handmade explosive charges to advance in the tunnels.

The results of this study showed that the male miners with a normal spirometry had a better quality of life than miners with an abnormal spirometry, specifically in physical function and physical role [1]. These results suggested that the miners from San Martin de Loba have worked in ASGMs and performed activities in tunnels extracting rocks containing gold-rich ores and later used hammers and ball mills to reduce the mineral to a fine powder. In addition, the miners use excavation techniques such as explosives, pneumatic, manual excavation tools, and work in poorly ventilated environments with low ventilation which are associated with dust or PM (Figure 4). Airborne PM has a strong association with lung capacity, reduced lung function, and pneumoconiosis (Figure 4). However, lung function may have a long impact if the miners are exposed to dust or PM with an aerodynamic diameter ≤ 2.5 μm (PM 2.5) [49,50].

In this study, 60% of the miners from San Martin de Loba had neurotoxic abnormalities (≥6) according to the Q16 neurological toxicity test. In addition, miners had 8.9 times more neurotoxic abnormalities (≥6) than non-miners. Therefore, miners with neurotoxic abnormalities (≥6) should be referred to higher levels of health care. These results were significant and were also found in adjusted analyses, including our small sample size. The present analysis also shows significant increases of some neurological parameters in miners, such as loss of understanding of TV/radio, problems with usual activities, fatigue, oppression in chest, painful tingling, loss of strength in arms, legs, loss of sensitivity in arms, legs and having more than 6 abnormalities, which guides us to advance with more specific neurological studies.

In Latin America, ASGM activities play an important role in affecting the quality of life of many communities. Dysfunctional households end up being a burden for women in these settings [8]. In San Martin de Loba, ASGM mining is the main economic activity, starting at an early age. It may cause school dropouts and increase addiction in the young population. Diet is also a relevant factor that depends on the consumption of fish from the Magdalena basin, adjacent to this town [4]. This body of water receives mercury contamination from ASGM activities and the nearby communities are affected by methylmercury-fish consumption. An earlier study in the San Martin de Loba showed that 90% of the participants eat fish approximately three to five times a week [24], potentially increasing the amount of Hg ingested in these mining sites. Based on the results of this study, further research is needed to evaluate how ASGM impacts lung function by measuring airborne particles such as PM2.5 and PM10 with a larger sample size and more detailed surveys to assess multiple stressors on respiratory health. Additionally, studies with larger sample sizes studies are needed to evaluate the health impact in miners as well as the impact on their communities.

## 5. Conclusions

ASGM is the primary source of employment or income for mining communities in Colombia and other countries, although it has raised concerns due to the effect of mercury pollution impacting the environment, and the health of miners and their families, including children and pregnant women. The exposure of miners to vapor and dust in ASGM activities has impacts on public health and on the environment. This study showed evidence of the association between mercury concentrations in blood samples from miners and neurotoxicity outcomes. In addition, it was observed that a significant association between spirometry results and quality of life in miners could be caused by the exposure to dust produced by limestone mining. This is the first report in Latin America that related the pulmonary function of miners working in ASGM sites. Spirometry patters as a pulmonary function test may be used as a bioindicator of the health status in an ASGM workplace and can be an important tool for health surveillance programs. Government, via the environmental and health agencies, must pay close attention to the mining population, because ASGM is characterized as a health risk and it lacks safety equipment and training for miners.

## Figures and Tables

**Table 1:** Characteristics of the surveyed population from San Martin de Loba, Colombia.

n	Total	Male		Female		*p*-Value
		Miners	Non-Miners	Miners	Non-Miners	
	124	89	9	17	9	
Age (years), median (range)	45.0 (37.0–53.2)	44.0 (37.0–58.0)	46.0 (39.0–53.0)	49.0 (34.0–58.0)	39.0 (31.0–48.0)	0.480
Body–mass index (kg/m^2^) median (range)	24.7 (16.9–36.0)	24.2 (23.1–29.7)	24.7 (22.9–36.0)	22.4 (16.9–27.3)	24.9 (21.8–27.1)	0.596
Work per day (h), median (range)	8 (6–10)	8 (6–8)	8.00 (8–10)	9 (8–10)	10 (8–12)	0.631
Time mining (years), median (range)	20 (2.0–50.0)	20 (3.0–30.0)	NA	11.0 (6.8–18.5)	NA	–
Education, n (%)						0.082
None	7.3	7.8	0	17.8	11.1	
Elementary	50.0	50.6	22.2	35.4	55.6	
High school	37.9	35.1	55.6	41.0	22.2	
Undergraduate degree	4.8	1.1	22.2	5.8	11.1	
Whole–blood T–Hg (μg/L) mean ± SD median (range)						**0.011**
	8.4 ± 10.0	8.6 ± 10.6	4.7 ± 2.9	10.2 ± 8.5	6.7 ± 10.4	
	6.0 (0.6–82.5)	6.2 (0.6–82.5)	4.6 (0.9–9.7)	8.5 (1.2–29.9)	2.40 (0.8–34.4)	
Whole blood T–Hg, n (%)						0.196
<5 μg/L	41.1	31.7	68.3	35.4	64.5	
5–15 μg/L (Environmental level)	10.5	10.3	2.4	11.2	5.6	
≥15 μg/ L (Occupational level)	26.4	16.9	3.3	50.9	33.3	

Notes: The percentages are calculated taking the fraction of the parameter in each column (miners and non-miners). Bold *p*-value numbers are significant at 0.05. SD: Standard deviation.

**Table 2:** Frequency of neurotoxic symptoms in the population surveyed in San Martin de Loba (Colombia).

Neurotoxic Symptoms (%)	Total	Miners	Non-Miners	*p*-Value
n	124	106	18	
Short memory	49.6	49.5	50.0	1.000
Family relates short memory	45.5	46.7	38.9	0.722
Forgets activities	38.2	38.1	38.9	1.000
Loss of meaning of TV/radio	30.1	31.4	22.2	0.611
Concentration	40.7	41.0	38.9	1.000
Irritation	54.5	52.4	66.7	0.385
Depressed	34.1	33.3	38.9	0.849
Problems with usual activities	34.1	34.3	33.3	1.000
Tired	63.4	66.7	44.4	0.123
Oppression in chest	49.6	55.2	16.7	**0.006**
Feeling of falling down when standing	37.4	38.1	33.3	0.903
Painful tingling	61.0	59.0	72.2	0.425
Buttoning test	19.5	19.0	22.2	0.752
Loss of strength in arms/legs	52.8	55.2	38.9	0.304
Loss of sensitivity in arms/legs	48.8	50.5	38.9	0.513
Problems sleeping	52.8	49.5	72.2	0.127
Abnormalities (≥6)	58.5	60.0	50.0	0.591

Note: Bold *p*-values numbers are significant at 0.05. IQR: interquartile range. The percentages were calculated by taking the fraction of the parameter in each column (miners and non-miners).

**Table 3:** Crude and adjusted effects of mercury levels as a risk factor for worse neurotoxic symptoms (Q16) in artisanal and small-scale miners from San Martin de Loba, Colombia.

Neurotoxic Effects (Q16) (%)	Miners		Non-Miners		Adjusted Odds Ratio *
	Low T-Hg	High T-Hg	Low T-Hg	High T-Hg	
n	40	66	11	7	
Short memory	45.0	52.3	54.5	42.9	3.3 (0.7–21.0)
Family relates short memory	35.0	53.8	45.5	28.6	3.9 (0.9–22.3)
Forgets activities	30.0	43.1	36.4	42.9	3.7 (0.8–26.0)
Loss of meaning of TV/radio	27.5	33.8	9.09	42.9	**20.4 (2.5–476.7)**
Concentration	30.0	47.7	36.4	42.9	1.5 (0.34–6.6)
Irritation	47.5	55.4	72.7	57.1	0.8 (0.2–4.2)
Depressed	30.0	35.4	45.5	28.6	4.8 (0.9–37.3)
Problems with usual activities	30.0	36.9	27.3	42.9	**8.2 (1.3–87.2)**
Tiredness	67.5	66.2	36.4	57.1	**233.2 (13.6–6725.1)**
Oppression in chest	62.5	50.8	9.09	28.6	**18.1 (3.03–154.8)**
Feeling of falling down when standing	40.0	36.9	27.3	42.9	**8.3 (1.4–96.0)**
Painful tingling	52.5	63.1	81.8	57.1	**0.8 (0.2–4.1)**
Buttoning test	12.5	23.1	18.2	28.6	1.6 (0.3–10.2)
Loss of strength in arms/legs	50.0	58.5	36.4	42.9	**11.5 (2.1–108.5)**
Loss of sensitivity in arms/legs	40.0	56.9	36.4	42.9	**9.3 (1.7–80.9)**
Problems sleeping	57.5	44.6	63.6	85.7	1.1 (0.2–8.8)
Abnormalities (≥6)	57.5	61.5	45.5	57.1	**8.9 (1.5–95.7)**

Low concentration T-Hg (Low): Hg < 5 μg/L, High concentration T-Hg (High): Hg ≥ 5 μg/L. * Odd ratios in logistic regression represent the likelihood that a neurotoxic effect (Q16) will take place which was adjusted by T-Hg concentration, age, sex, body mass index, and occupation. Bold *p*-value numbers are significant at ≤0.05.

**Table 4:** Concentrations of T-Hg as a risk factor for lower quality of life (SF-36), in miners from San Martin de Loba, Colombia.

Quality of Life Parameter (%)	Total	High T-Hg		Low T-Hg		*p*-Value
		Female	Male	Female	Male	
n	124	9	16	17	82	
Physical function	85.0 (50–95)	50 (25–75)	85 (60–93)	55 (40–85)	90 (75–100)	**0.003**
Physical role	50.0 (0–100)	50 (0–100)	50 (0–100)	0 (0–75)	75 (12–100)	0.165
Pain	71.0 (41–90)	41 (30–64)	73 (38–85)	32 (22–51)	74 (51–90.0)	**0.005**
General health	57.0 (45 –70)	42 (40–55)	60.0 (48–75)	45.0 (35–50)	62 (50–70.5)	**0.003**
Vitality	70.0 (55–90)	50 (25–60)	70 (52–85)	45 (30–65)	80 (65–90)	**<0.001**
Social function	87.5 (62–100)	50 (37–88)	81 (68–100)	62 (37–100)	100 (75–100)	**0.006**
Emotional role	86.0 (33–100)	76 (42–100)	79 (33–100)	84 (45–100)	87(33–100)	**0.042**
Mental health	80.0 (64–96)	56 (44–60)	92 (80–96)	64 (32–88)	84 (76–99)	**0.001**

Note: Continuous variables report median and interquartile range (25th and 75th percentile). Bold *p*-values numbers are significant at 0.05. Low concentrations of T-Hg (Low): Hg < 5 μg/L, High concentrations of T-Hg (High): Hg ≥ 5 μg/L.

**Table 5:** Respiratory capacity and Quality of life survey in male miners from San Martin de Loba, Colombia (*n* = 87).

Parameter	Normal Spirometry	Abnormal Spirometry		Obstructive Spirometry		Restrictive Spirometry	
	Median (IQR)	Median (IQR)	*p*-Value	Median (IQR)	*p*-Value	Median (IQR)	*p*-Value
n	65	22		66		16	
Physical function	95.0 (80.0–100)	75.0 (45.0–86.2)	**0.001**	60.0 (33.8–82.5)	0.029	75.0 (55.0–88.8)	**0.004**
Physical role	87.5 (25.0–100)	12.5 (0.00–68.8)	**0.003**	25.0 (0.00–50.0)	0.059	12.5 (0.00–81.2)	**0.012**
Pain	74.0 (51.0–90.0)	62.0 (22.0–90.0)	0.298	81.0 (34.5–90.0)	0.898	52.0 (21.5–87.0)	0.177
General health	62.0 (52.0–72.0)	57.5 (45.0–67.0)	0.310	47.5 (41.2–57.5)	0.129	61.0 (48.8–68.2)	0.714
Vitality	80.0 (65.0–90.0)	70.0 (60.0–85.0)	0.360	80.0 (65.0–83.8)	0.822	65.0 (60.0–87.5)	0.321
Social function	100 (75.0–100)	87.5 (75.0–100)	0.159	75.0 (75.0–84.4)	0.176	87.5 (71.9–100)	0.338
Emotional role	100 (33.3–100)	66.7 (41.7–100)	0.359	66.7 (41.7–91.7)	0.365	83.3 (50.0–100)	0.543
Mental health	84.0 (76.0–96.0)	92.0 (82.0–98.0)	0.335	96.0(96.0–100)	0.266	90.0 (81.0–96.0)	0.589
Physical function	40.0 (20.0–40.0)	40.0 (20.0–55.0)	0.992	50.0 (40.0–60.0)	0.126	30.0 (15.0–40.0)	0.388

Note: Continuous variables report median and interquartile range (25th and 75th percentile). *p*-value numbers in bold are significant at 0.05. Out of the 22 abnormal spirometry results, 6 have obstructive patterns, and 16 have restrictive patterns.

**Table 6:** Systematic review of studies of Hg levels in mining communities of The Americas.

Country, Year	Miner Population (*n*)	Hair Mean (μg/g) (95% CI) [Range]	Blood Mean (μg/L) (95% CI) [Range]	Urine Mean (μg/L) (95% CI) [Range]	Ref.
Colombia, 2016	All sample (95 men and 105 women)	17.3 [1.2–47]			[5]
Colombia, 2017	Men (55)	3.29 [0.60–15.54]			[31]
	Women (26)	0.77 [0.06–1.80]			
	All sample (81)	2.48 [0.06–17.54]			
Colombia, 2018	Miners (Quibdo) (248)	1.26 [0.02–116.40]			[32]
	Miners (Paimado) (112)	0.67 [0.07–6.47]			
	Miners (Atrato River Basin) (360)	0.92 [0.02–116.40]			
Colombia, 2018	Miners (16)	15.98	11.29	23.89	[33]
	Women (19)	8.55	8.8	5.37	
	Dealers (23)	26.79	11.7	32.53	
	Girls	4.45			
	Boys	1.11			
Colombia, 2018	Women (36)	2.3 (5.1–24)	7.6 (4.2–15.3)	14 (5.2–26.5)	[34]
	Miners (451)	2.1 (5.2–26.5)	7.4 (3.6–14.8)	9.9 (5.1–24)	
Colombia, 2019	All sample (women 30 and man 20)		21.9 (3.1–0.7)		[27]
Colombia, 2019	All sample (522)	3.07 (0.15–25.1)			[35]
Colombia, 2020	All samples (men 101 and women 59)	0.8 * (0.5–1.3 **)	7 * [3.4–11 **]	3.9 * (1.3–4.1 **)	[36]
Colombia, 2021	Women (203)	3.1 (0.7–5.6)	4.9 (1.7–16.7)	4.7 (1.9–14.1)	[37]
	Men (35)	1.4 (0.5–4.4)	3.6 (1.4–12.2)	3.8 [2.9–10.1 **]	
Ecuador, 2016	Men (44)			50.8 [15–163]	[38]
México, 2016	Women (3)			39.7 [20.2–63.4]	[39]
	Men (8)			54.1 [11.5–144]	
México, 2021	Men (BUC) (17)			51.5 [0.8–51.5] ***	[40]
	Men (LSF) (14)			105.9 [9.9–09.7] ***	
	Women (BUC) (17)			2.8 [1.7–4.9] ***	
	Women (LSF) (14)			4.7 [1.3–10.1] ***	
Peru, 2017	Women (41)	2.1 (0.3–11.0)			[31]
	Men (39)	2 (0.3–11.0)			
	All simple (80)	2 (0.5–10.1)			
Peru, 2019	Women (200)	1.6 [0.01–30.12]			[41]

Note: This study carried out an exhaustive review of the literature, initially finding 221 studies. After refining the search, 15 studies of Hg exposure in artisanal miners in the Americas were chosen. * This is the median estimation of Hg levels. ** This is the interquartile range. *** μg/g creatinine (urine sample).

## Data Availability

The data are available upon reasonable request to the corresponding author.
